# Comparison of the Safety and Efficacy of Laparoscopic Left Lateral Hepatectomy and Open Left Lateral Hepatectomy for Hepatolithiasis: A Meta-Analysis

**DOI:** 10.3389/fsurg.2021.749285

**Published:** 2021-11-16

**Authors:** Xiaoji Wang, Ai Chen, Qiurong Fu, Chunping Cai

**Affiliations:** ^1^Department of Liver and Gallbladder Surgery, The First Affiliated Hospital of Hainan Medical University, Haikou, China; ^2^Department of Nursing, The First Affiliated Hospital of Hainan Medical University, Haikou, China

**Keywords:** laparoscopic left lateral hepatectomy, open left lateral hepatectomy, safety, efficacy, meta-analysis

## Abstract

**Background:** Intrahepatic duct (IHD) stones, also known as hepatolithiasis, refers to any intrahepatic stones of the left and right hepatic ducts. It is a benign biliary tract disease with a high recurrence rate, with many complications, and difficulty in radical cure. The aim of this review and meta-analysis is to compare the safety and efficacy of the laparoscopic left lateral hepatectomy (LLLH) and open left lateral hepatectomy (OLLH) for IHD stones.

**Methods:** Pubmed, Embase, Cochrane, WangFang Data, and China National Knowledge Infrastructure were searched for randomized controlled trials (RCTs) regarding the comparison of LLLH and OLLH in the treatment of hepatolithiasis. Standard mean difference (SMD), odds ratio (OR), and 95% CI were calculated using the random-effects model or fixed-effects model according to the heterogeneity between studies.

**Results:** From January 01, 2001 to May 30, 2021, 1,056 articles were retrieved, but only 13 articles were finally included for the meta-analysis. The results showed that compared to the OLLH group, LLLH resulted in smaller surgical incision, less intraoperative blood loss, faster postoperative recovery, and fewer postoperative complications (surgical incision: SMD = −3.76, 95% CI: −5.40, −2.12; intraoperative blood loss: SMD = −0.95, 95% CI: −1.69, −0.21; length of hospital stay: SMD = −1.56, 95% CI: −2.37, −0.75; postoperative complications: OR = 0.45, 95% CI: 0.26, 0.78).

**Conclusions:** In the treatment of hepatolithiasis, compared with OLLH, LLLH has the advantages of less intraoperative blood loss, smaller incisions, less postoperative complications, shorter hospital stay, shorter time to first postoperative exhaust, and postoperative ambulation, and rapid postoperative recovery.

## Introduction

Intrahepatic duct (IHD) stones, also known as hepatolithiasis, refers to any intrahepatic stones of the left and right hepatic ducts. It is a benign biliary tract disease with a high recurrence rate, many complications, and difficulty in radical cure (1). IHD stones are often distributed in the hepatic segments and lobes, especially in the left lateral lobe and right posterior lobe of the liver. Due to the poor biliary drainage caused by the anatomical variations of the confluence of the bile duct of these two lobes and the common bile duct, IHD stones easily enter the common bile duct and lead to secondary choledocholithiasis. The causes of IHD stones are relatively complex and related to biliary tract infection, cholestasis, and biliary parasites. Recurrent infection of hepatolithiasis can cause local liver atrophy, fibrosis, and loss of function. This disease is mainly prevalent in Asia–Pacific countries, and is related to dietary habits and nutritional status in this area (1, 2). At present, the treatment of hepatolithiasis is mainly based on the principle of “removing lesions, extracting stones, correcting stricture, maintaining unobstructed drainage, and preventing recurrence” (3).

Open hepatectomy has been widely used in clinical practices because of its ease of operation. However, it is relatively invasive to patients and the postoperative recovery is slow (4). The continuous progress of laparoscopic hepatectomy promotes the application of selective portal inflow occlusion, and therefore this method has gradually become an important means of treating hepatolithiasis. However, laparoscopic hepatectomy is still associated with a high risk of hemorrhage and high procedural difficulty, and its clinical efficacy and safety are still controversial (4). Therefore, the aim of this review and meta-analysis is to compare the safety and efficacy of laparoscopic left lateral hepatectomy (LLLH) and open left lateral hepatectomy (OLLH) for IHD stones.

## Methods

The systematic review followed the methodology outlined in the Cochrane Handbook for Systematic Reviews of Interventions Version 6.0 (5). This study was reported based on the PRISMA-P (Preferred Reporting Items for Systematic Reviews and Meta-Analyses Protocols) (6).

### Search Strategy

PubMed, Embase, Cochrane, WangFang Data, and the China National Knowledge Infrastructure were searched for clinical randomized controlled trials (RCTs) on LLLH and OLLH in the treatment of hepatolithiasis from January 01, 2001, to May 31, 2021. The search items were as follows: ((intrahepatic duct stones) OR (intrahepatic duct stones [MeSH Terms]) OR (hepatolithiasis) OR (hepatolithiasis [MeSH Terms])) AND ((left lateral hepatectomy) OR (left lateral hepatectomy [MeSH Terms])) AND ((laparoscopic) OR (laparoscopic [MeSH Terms]) OR (open) OR (open [MeSH Terms])). In addition, the references of the preliminarily included articles, which were eligible for full-text article assessment, were also systematically searched for preventing omission and comprehensively comparing the safety and efficacy of LLLH and OLLH in the treatment of hepatolithiasis.

### Inclusion and Exclusion Criteria

Two researchers independently assessed the titles and abstracts of the studies obtained from the initial search according to the inclusion and exclusion criteria. During this process, the disagreement was resolved by consulting a third researcher who would determine whether to finally include a trial based on the opinion of the former two. Inclusion criteria incorporated the elements included in the PICOS protocol, as follows: (1) participants: patients with hepatolithiasis. (2) intervention and comparison: the surgical methods were LLLH and OLLH, and a comparison of the safety and efficacy of these two surgical methods was provided. (3) outcomes: operation time, intraoperative blood loss, length of hospital stay, postoperative complications, and other indicators. (4) Study design: only RCTs were included to ensure that the pooled results were of good quality. Exclusion criteria were as follows: (1) The full text could not be obtained or the required data could not be extracted from the full text; (2) the outcome measures of the two surgical methods were not provided; (3) duplicated publication of the same trial; (4) the report data were incomplete and the relevant data could not be obtained from reasonable channels; (5) with major deficiencies in study design or major biases in the reporting of results. Studies that met any of the above criteria were excluded.

### Data Extraction and Quality Assessment

The two researchers independently extracted the following data provided by each included study: title, first author, publication year and journal, number of included study subjects, grouping, age, inclusion and exclusion criteria, operation time, intraoperative blood loss, stone clearance rate, length of hospital stay, postoperative complications, and study design-related indicators (mainly including study protocol and quality control). After data extraction, the third researcher checked the consistency of the data extracted by the former two researchers.

The quality of the included RCTs was independently evaluated by the two researchers according to the RCT quality assessment section of the Cochrane handbook for systematic reviews of interventions 6.0 (5). The articles that met the evaluation items were included in the final systematic review and meta-analysis. Specifically, for each included study, the two researchers assessed blind bias, incomplete outcome bias, selective reporting bias, selection bias, and other biases. During this process, the disagreement was resolved by the third researcher who made a final decision based on the opinion of the former two. Finally, according to the Cochrane handbook, the included studies were divided into low, medium, and high risk.

### Statistical Analysis

Results were merged across studies with STATA version 15.1 (Stata Corp MP., College Station, TX, USA) (7, 8). Study subjects in each included study were patients with hepatolithiasis who received LLLH or OLLH, and the comparison of the two surgical methods was provided, suggesting a good consistency. Assessment of heterogeneity was performed using the Q test and *I*^2^ statistics. *I*^2^ values of 0–39%, 40–59%, and 60–90% indicated low, moderate, and high heterogeneity among studies, respectively (5). In case of low heterogeneity, the fixed-effects model was adopted for pooling the results, otherwise, the random-effects model was employed. For each dichotomous variable, OR and its 95% confidence interval (CI) were utilized to compare the safety of LLLH and OLLH in the treatment of hepatolithiasis, whereas for each continuous variable, standard mean difference (SMD) and 95% CI were used. If the number of studies comparing the safety and efficacy of the two surgical methods was ≥ 5, the results were presented as forest plots, otherwise the results were presented in tables. If the number of studies was ≥ 5, the Egger's test was used for assessing the publication bias of the results and Duval and Tweedie's trim and fill test for the sensitivity of the results (9, 10). Exact *P*-values would be reported unless *P* < 0.001. *P* < 0.10 in the result of Egger's test was considered statistically significant and significant differences were suggested in other results if *P* < 0.05.

## Results

### Literature Search, Study Characteristics, and Quality Assessment

**Figure 1:**
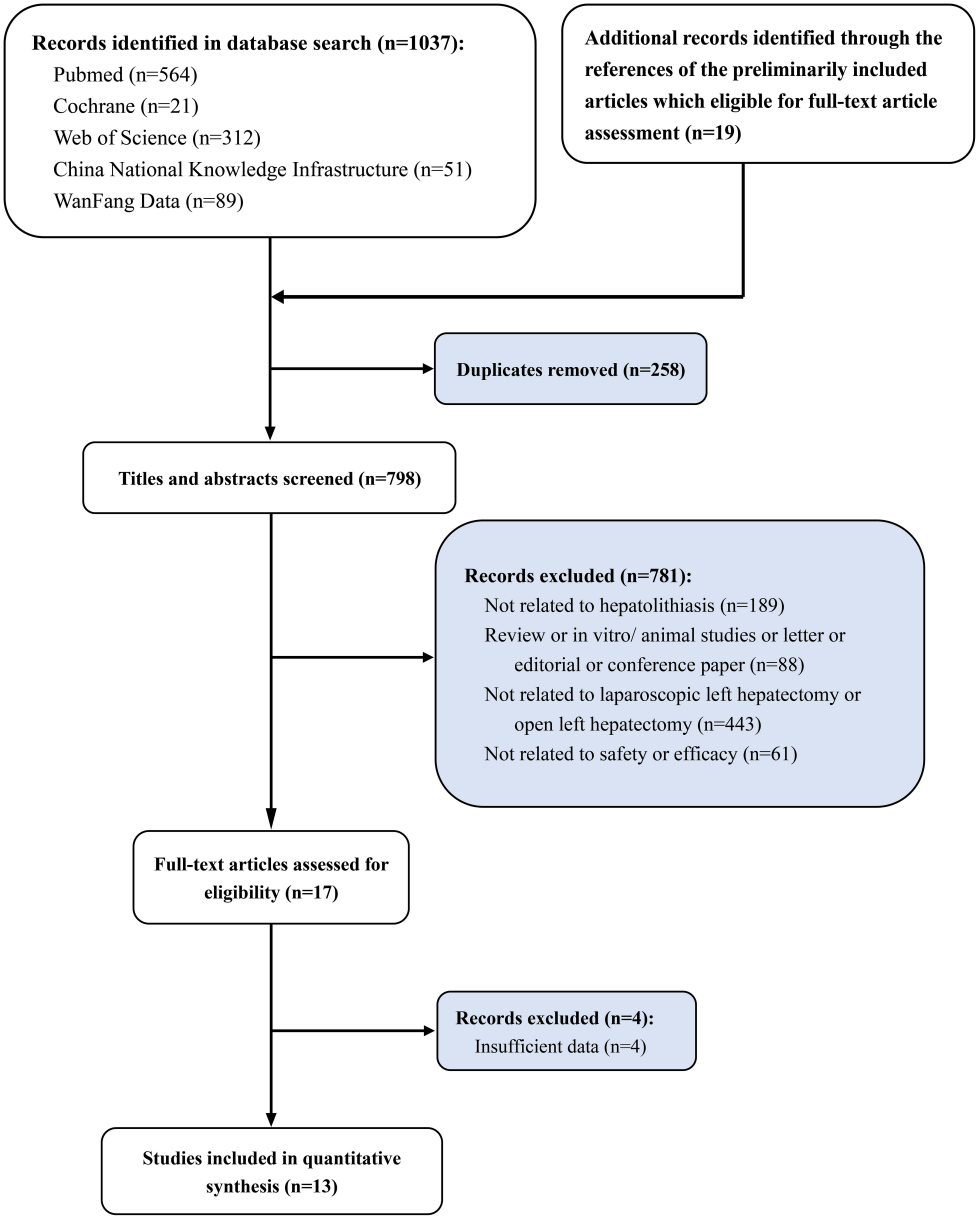
Study selection flowchart, systematic review, and meta-analysis of comparison of the safety and efficacy of laparoscopic left lateral hepatectomy and open left lateral hepatectomy for hepatolithiasis.

A total of 1,037 articles were obtained by database retrieval and 19 articles by searching the references of the preliminarily included articles which were eligible for full-text article assessment. On completion of exclusion of 258 duplicate articles, 781 were then excluded on the basis of titles and abstracts (not related to hepatolithiasis, *n* = 189; review or *in vitro*/animal studies or letter or editorial or conference paper, *n* = 88; not related to laparoscopic left hepatectomy or open left hepatectomy, *n* = 443; not related to safety or efficacy, *n* = 61). On reading the full texts, four articles without valid data were excluded, and therefore 13 studies were finally included in the meta-analysis (11–23) (Figure 1), including 542 patients with hepatolithiasis treated with LLLH and 531 treated with OLLH. The basic characteristics of the 13 included RCTs are shown in Table 1.

**Table 1:** Baseline characteristics of included studies for meta-analysis.

**References**	**No. of cases**		**Average age**	**Detail of hepatolithiasis**	**Detail of surgery**
	**LLLH**	**OLLH**			
Ding et al. (11)	49	49	57.53 ± 6.31	Maximum size of hepatolithiasis: open group (0.97 ± 0.21) vs. laparoscopic group (0.96 ± 0.26); No. hepatolithiasis (3 or more): open group (7) vs. laparoscopic group (5);	Left lateral lobectomy which only include segments 2 and 3.
Wu et al. (12)	36	36	54.44 ± 3.78	Open group: left intrahepatic bile duct stone (7 cases) + left intrahepatic bile duct stone combined with choledocholithiasis (16 cases) + left intrahepatic bile duct stone combined with right intrahepatic bile duct stone (7 cases) + left intrahepatic bile duct and common bile duct and right intrahepatic bile duct calculi (6 cases); laparoscopic group: left intrahepatic bile duct stone (8 cases) + left intrahepatic bile duct stone combined with choledocholithiasis (15 cases) + left intrahepatic bile duct stone combined with right intrahepatic bile duct stone (6 cases) + left intrahepatic bile duct and common bile duct and right intrahepatic bile duct calculi (7 cases)	Left hepatectomy which include segments 2, 3, and 4. Stones located in the right bile duct or main bile duct will be explored and removed from the left bile duct section.
Li (13)	30	30	43.80 ± 1.30	Open group: Child category (A: 12 cases; B: 18 cases); laparoscopic group: Child category (A: 11 cases; B: 19 cases)	Left lateral lobectomy which only include segments 2 and 3.
Huangfu (14)	38	38	49.20 ± 11.78	No description	Left lateral lobectomy which only include segments 2 and 3.
Dong (15)	43	43	43.24 ± 11.22	No description	Left lateral lobectomy which only include segments 2 and 3.
Chen et al. (16)	46	37	42.10 ± 4.30	Open group: Child category (A: 28 cases; B: 9 cases); laparoscopic group: Child category (A: 34 cases; B: 12 cases)	Left lateral lobectomy which only include segments 2 and 3.
Li et al. (17)	34	34	49.67 ± 11.64	No description	Left lateral lobectomy which only include segments 2 and 3.
Li (18)	57	57	56.82 ± 8.66	Open group: Child category (A: 42 cases; B: 15 cases); laparoscopic group: Child category (A: 40 cases; B: 17 cases)	Left lateral lobectomy which only include segments 2 and 3.
Yao et al. (19)	57	57	53.27 ± 7.62	No description	Left hepatectomy which include segments 2, 3, and 4.
Wang et al. (20)	62	60	38.70 ± 1.50	No description	Left hepatectomy which include segments 2, 3 and 4.
Xie (21)	40	40	51.26 ± 4.43	No description	Left lateral lobectomy which only include segments 2 and 3.
Zhou (22)	29	29	45.28 ± 3.69	No description	Left lateral lobectomy which only include segments 2 and 3.
Sun (23)	21	21	55.80 ± 5.00	No description	Left hepatectomy which include segments 2, 3, and 4.

*LLLH, laparoscopic left lateral hepatectomy; OLLH, open left lateral hepatectomy*.

**Figure 2:**
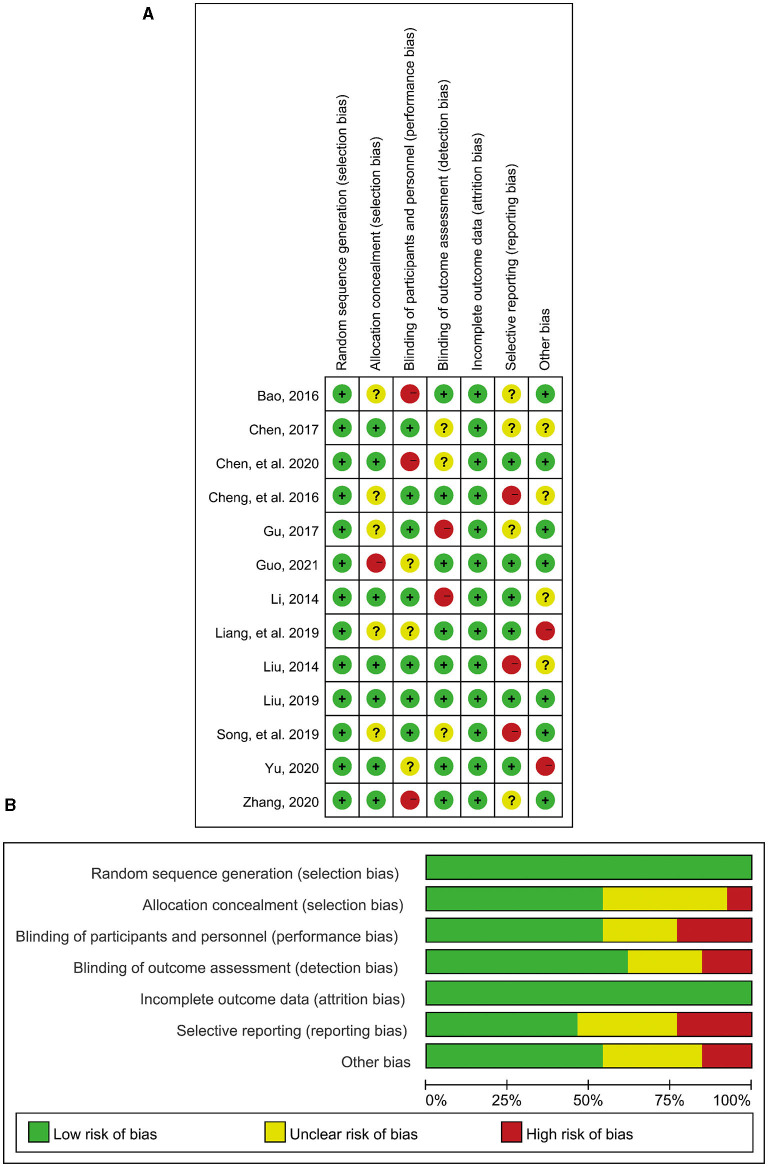
Literature quality assessment. **(A)** Risk of bias summary: Review judgments of authors about each risk of bias item for each included study; **(B)** Risk of bias graph: Review judgments of authors about each risk of bias item presented as percentages across all included studies.

In terms of quality assessment, all the included studies strictly followed the principle of random allocation, and patients with the possibility of unpredictable adverse caused by the trials were all excluded before the investigation in each study. Therefore, there was no incomplete reporting bias in the included studies, causing no damage to the power of the test. Both biases were assessed as low risk. Collectively, the overall assessment of the included RCTs considered a low risk of bias, indicating good quality of this meta-analysis and high reliability of the results (Figures 2A,B).

### Results of Meta-Analysis

#### Comparison Between LLLH and OLLH in Hepatolithiasis Patients

**Figure 3:**
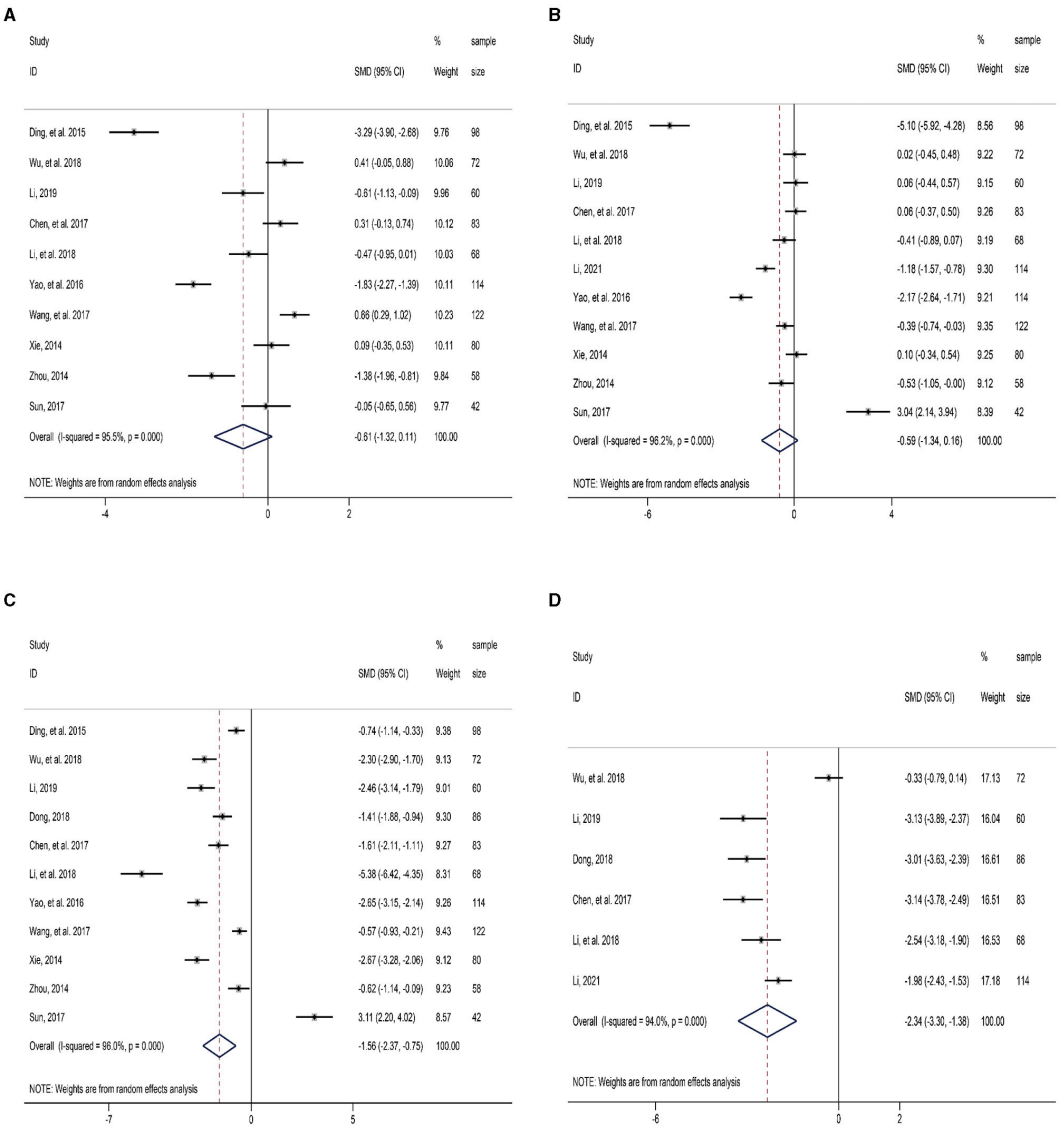
Forest plot of comparison between LLLH and OLLH in hepatolithiasis patients. **(A)** operation time; **(B)** intraoperative blood loss; **(C)** length of hospital stay; and **(D)** first postoperative flatus.

Ten RCTs reported the operation time required for LLLH and OLLH in the treatment of hepatolithiasis. The results of the meta-analysis showed that LLLH required less operation time than OLLH, but the difference was not statistically significant (SMD = −0.61, 95% CI: −1.32, 0.11; Figure 3A and Table 2). Notably, due to the strong heterogeneity of this indicator among the studies (*I*^2^ = 95.5%), the guiding significance of this result required further discussion.

**Table 2:** Summarized results of included studies.

**Indicators**	**No. of studies**	**Sample size**	**Effect size (95%CI)**	**Heterogeneity (%)**	
				***I^**2**^***	***P***
**Comparison between LLLH and OLLH in hepatolithiasis patients**					
Operation time	10	797	−0.61 (−1.32, 0.11)	95.5	<0.001
Intraoperative blood loss	11	911	−0.59 (−1.34, 0.16)	96.2	0.001
Length of hospital stay	11	883	−1.56 (−2.37, −0.75)	96.0	<0.001
First postoperative flatus	6	483	−2.34 (−3.30, −1.38)	94.0	<0.001
Time to postoperative ambulation	5	415	−3.44 (−5.26, −1.63)	97.1	<0.001
Surgical incision length	5	409	−3.76 (−5.40, −2.12)	96.1	<0.001
Postoperative complications	12	959	0.45 (0.26, 0.78)	50.1	0.024
Residual stones	4	387	0.99 (0.40, 2.45)	0.0	0.951

*CI, confidence interval; LLLH, laparoscopic left lateral hepatectomy; OLLH, open left lateral hepatectomy*.

Eleven RCTs reported intraoperative blood loss. The results of the meta-analysis revealed that LLLH had less intraoperative blood loss than OLLH in the treatment of hepatolithiasis, but the difference was also not statistically significant (SMD = −0.59, 95% CI: −1.34, 0.16; Figure 3B and Table 2). Similarly, the heterogeneity of this indicator was as high as 96.2%.

Eleven studies reported the length of hospital stay. The patients treated with LLLH had a shorter hospital stay than those treated with OLLH, and the difference was statistically significant (SMD = −1.56, 95% CI: −2.37, −0.75; Figure 3C and Table 2). There was strong heterogeneity among the studies (*I*^2^ = 96.0%, *P* < 0.001).

Six RCTs reported the time till the first postoperative flatus. The first postoperative exhaust time was significantly earlier in the LLLH group than in the OLLH group (SMD = −2.34, 95% CI: −3.30, −1.38; Figure 3D and Table 2). The source of the strong heterogeneity found by sensitivity analysis might be related to the study by Wu et al. (12).

**Figure 4:**
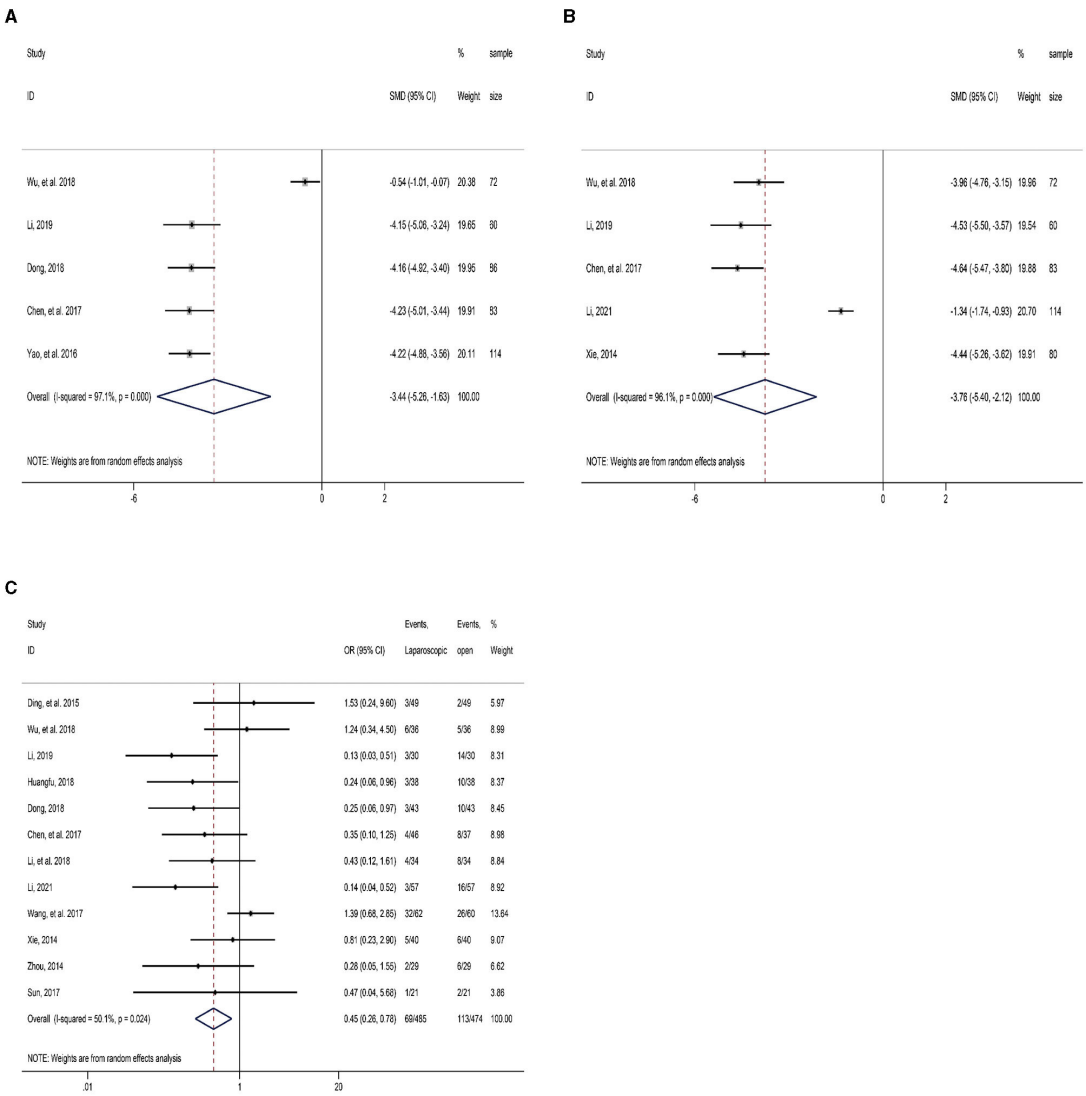
Forest plot of comparison between LLLH and OLLH in hepatolithiasis patients. **(A)** time to postoperative ambulation; **(B)** surgical incision length; and **(C)** postoperative complications.

Five RCT studies reported the time to postoperative ambulation. The meta-analysis results demonstrated that the time till postoperative ambulation was markedly earlier in the LLLH group than that in the OLLH group (SMD = −3.44, 95% CI: −5.26, −1.63; Figure 4A and Table 2). Strong heterogeneity among studies was identified, and coincidentally, sensitivity analysis found that its source was also associated with the study by Wu et al. (12).

Five studies reported the surgical incision length. The result determined that the incision length in the LLLH group was significantly shorter than that in the OLLH group (SMD = −3.76, 95% CI: −5.40, −2.12; Figure 4B and Table 2). Sensitivity analysis indicated that the strong heterogeneity of this indicator might be related to the study by Li (18).

Twelve RCTs reported postoperative complications. Moderate heterogeneity among the studies (*I*^2^ = 50.1%, *P* = 0.024) was identified, and so the random-effects model was utilized. The result showed a significantly lower incidence of postoperative complications in the LLLH group in comparison with the OLLH group (OR = 0.45, 95% CI: 0.26, 0.78; Figure 4C and Table 2).

Four RCT studies reported residual stones after surgery. There was no heterogeneity among the studies (*I*^2^ = 0.0%, *P* = 0.95), and so the fixed-effects model was used to pool the results. According to the result, no significant difference was identified in postoperative residual stones between the two groups (OR = 0.99, 95% CI: 0.40, 2.45; Table 2).

#### Publication Bias Assessment and Sensitivity Analysis

We used Egger's test to analyze the publication bias of each indicator. With small-study effects, the test result found publication bias in the incision length (*P* < 0.10), while no significant publication bias was observed in other indicators (Table 3). The Duval and Tweedie's trim and fill test for hemorrhage volume revealed that its effect size was not stable, and the difference of this indicator changed from the original non-statistical significance to statistical significance after trimming and filling (Table 3). Therefore, the guiding significance of this indicator was required for further discussion.

**Table 3:** Evaluation of publication bias and sensitivity analysis.

**Index**	**Egger's regression**		**Duval and Tweedie's trim and fill**		
	**Intercept**	***p***	**Original effect size**	**Studies trimmed**	**Adjusted effect size**
Operation time	−15.057	0.167	−0.61 (−1.32, 0.11)	0	−0.61 (−1.32, 0.11)
Intraoperative blood loss	−1.504	0.838	−0.59 (−1.34, 0.16)	2	−0.95 (−1.69, −0.21)
Length of hospital stay	−4.851	0.415	−1.56 (−2.37, −0.75)	0	−1.56 (−2.37, −0.75)
First postoperative flatus	−14.883	0.178	−2.34 (−3.30, −1.38)	0	−2.34 (−3.30, −1.38)
Time to postoperative ambulation	−20.355	0.036	−3.44 (−5.26, −1.63)	0	−3.44 (−5.26, −1.63)
Surgical incision length	−13.166	0.002	−3.76 (−5.40, −2.12)	0	−3.76 (−5.40, −2.12)
Postoperative complications	−1.572	0.259	0.45 (0.26, 0.78)	0	0.45 (0.26, 0.78)

## Discussion

Hepatolithiasis is a common form of stone disease, especially in Southeast Asia (1). Although belonging to the category of benign lesions, this disease is easy to lead to bile duct dilatation, resulting in stenosis and ultimately induce biliary tract infection, liver parenchymal atrophy, and even cholangiocarcinoma. IHD stones are characterized by a complex condition, long course of the disease, and easy recurrence, which threatens the life and health of patients if not cured effectively (24). The stones mostly locate in the left lateral lobe, so in the treatment of hepatolithiasis, left hepatectomy is more common than right hepatectomy. The former can not only remove stones but also treat biliary stricture. For a long time, OLLH has been the main method for hepatolithiasis, but it has the risks of incision infection, liquefaction, dehiscence, unaesthetic, long exposure time of the organs, and slow postoperative recovery (25, 26). Since the first report of successful application of laparoscopic hepatectomy in liver resection in 1991, this method has been widely used for liver lesions, including benign and malignant types (27). In the past 20 years, laparoscopic operation and relevant devices have been continuously improved. Laparoscopic hepatectomy has the advantages of the short operation time, rapid postoperative recovery, less trauma, and less postoperative complications, and its feasibility and safety have also been confirmed by several large-scale studies (28, 29). In this systematic review and meta-analysis, we focused on the comparison of the safety and efficacy of LLLH and OLLH in the treatment of hepatolithiasis based on clinical RCT studies, and found that LLLH was more effective than OLLH.

There was no clinical significance in the difference of operation time between the LLLH and OLLH groups Because of two reasons. First, the result of the meta-analysis of this indicator had high heterogeneity, and no marked consistency could be observed (Figure 3A). Second, surgical operators in different studies had different proficiencies in surgery, and thus the basic conditions for the comparison for meta-analysis could not be provided. Therefore, we believe that the difference in the operation time has only statistical significance and no clinical significance.

Laparoscopic left lateral hepatectomy resulted in less hemorrhage during surgery in comparison with OLLH. Duval and Tweedie's trim-and-fill test on the studies by Ding et al. (11) and Sun (23) found that after trimming and filling, the difference in hemorrhage volume changed from non-statistical significance to statistical significance. Judging from the percentage of each study, after removing the variation indicator that has a great impact on the outcome, we consider that the result after trimming and filing reflects the real situation. That is, LLLH causes less hemorrhage in comparison with OLLH, and the difference had statistical significance (SMD = −0.95, 95% CI: −1.69, −0.21, Table 3).

In addition, the meta-analysis result of the incision length was affected by the small sample size of the studies. Observation on its forest plot confirmed that small studies regarding incision length were included in the meta-analysis, with 72, 60, 83, and 80 cases in the studies of Wu et al. (12), Li (13), Chen and Ou (16), and Xie (21), respectively. The sample size of these four studies was less than that of Li (13) (114 cases), but five studies were nearly equally divided into 20% of the final results of the meta-analysis. Therefore, it is normal for Egger's test to detect the small-study effects in this indicator. From the consistency of various study results, we can unquestionably determine that the total incision length of the LLLH is shorter than that of the OLLH group. Even if there was high heterogeneity in other indicators, according to the good consistency among the studies, we can conclude that LLLH is safer and more effective than OLLH in the treatment of hepatolithiasis. That is, LLLH is associated with less intraoperative blood loss and postoperative complications, shorter hospital stay, shorter time to first postoperative exhaust, and postoperative ambulation.

This study still has some limitations. First, the studies included in this review are mainly limited to the Chinese because this disease is more common in Southeast Asia. More multicenter and large-sample RCTs from the world are still required to further verify the efficacy and safety of LLLH in the treatment of hepatolithiasis so as to expand the application value of the conclusions. Second, the experience and surgical skills of different surgical operators vary from study to study, which have a certain impact on the surgical results and affect the stability and reliability of the conclusions. Third, there are four studied surgeries for left hepatectomy, which includes segments 2, 3, and 4. This would cause some bias and contribute to unobjective results for the safety and efficacy of LLLH and OLLH for hepatolithiasis. But, as we know, the difficulty of operation will not change much in left lateral hepatectomy with or without segment 4. So, that would contribute to heterogeneity, but it is acceptable and will provide more evidence to analyze the safety and efficacy of LLLH and OLLH for hepatolithiasis.

In conclusion, in the treatment of hepatolithiasis, compared with OLLH, LLLH has the advantages of less intraoperative blood loss, smaller incisions, less postoperative complications, shorter hospital stay, shorter time to first postoperative exhaust and postoperative ambulation, and rapid postoperative recovery.

## Data Availability Statement

The raw data supporting the conclusions of this article will be made available by the authors, without undue reservation.

## Author Contributions

XW, AC, and CC: critical revision of the manuscript, substantial contribution toward the conception and design of the work, and manuscript drafting. XW, AC, and QF: acquisition, analysis, and interpretation of the data. XW, AC, QF, and CP: revising the manuscript critically and final approval of the version to be published. All the authors have read and approved the final manuscript.

## Conflict of Interest

The authors declare that the research was conducted in the absence of any commercial or financial relationships that could be construed as a potential conflict of interest.

## Publisher's Note

All claims expressed in this article are solely those of the authors and do not necessarily represent those of their affiliated organizations, or those of the publisher, the editors and the reviewers. Any product that may be evaluated in this article, or claim that may be made by its manufacturer, is not guaranteed or endorsed by the publisher.

## Abbreviations

IHD: Intrahepatic Duct
LLLH: Laparoscopic Left Lateral Hepatectomy
OLLH: Open Left Lateral Hepatectomy
SMD: Standard Mean Difference
OR: Odds Ratio
CI: Confidence Interval
RCTs: Randomized Controlled Trials.
